# Status and trends of tundra birds across the circumpolar Arctic

**DOI:** 10.1007/s13280-019-01308-5

**Published:** 2020-01-18

**Authors:** Paul A. Smith, Laura McKinnon, Hans Meltofte, Richard B. Lanctot, Anthony D. Fox, James O. Leafloor, Mikhail Soloviev, Alastair Franke, Knud Falk, Mikhail Golovatin, Vasiliy Sokolov, Aleksandr Sokolov, Adam C. Smith

**Affiliations:** 1 Wildlife Research Division, Environment and Climate Change Canada, National Wildlife Research Centre, 1125 Colonel By Dr, Ottawa, ON K1S 5B6 Canada; 2 Department of Multidisciplinary Studies and Graduate Program in Biology, York University, Glendon Campus, 2275 Bayview Ave, Toronto, ON M5B 3M6 Canada; 3 Department of Bioscience, Aarhus University, Frederiksborgvej 399, 4000 Roskilde, Denmark; 4 Migratory Bird Management, U.S. Fish and Wildlife Service, 1011 East Tudor Road, Anchorage, AK 99503 USA; 5 Department of Bioscience, Aarhus University, Kalø, Grenåvej 14, 8410 Rønde, Denmark; 6 Canadian Wildlife Service, Environment and Climate Change Canada, 150-123 Main St, Winnipeg, MB R3C 4W2 Canada; 7 Department of Vertebrate Zoology, Lomonosov Moscow State University, Moscow, Russia 119991; 8 Department of Biological Sciences, University of Alberta, Edmonton, AB Canada; 9 www.vandrefalk.dk, Ljusstöparbacken 11A, 11765 Stockholm, Sweden; 10 Institute of Plant and Animal Ecology Ural Branch, Russian Academy of Sciences, 8 Marta Str, 202, Ekaterinburg, Russia 620144; 11 Arctic Research Station, Institute of Plant and Animal Ecology, Zelenaya Gorka Str., 21, Yamal-Nenets Autonomous District, Labytnangi, Russia 629400; 12 Canadian Wildlife Service, Environment and Climate Change Canada, 1125 Colonel By Dr, Ottawa, ON K1S 5B6 Canada; 13 Department of Biology, Carleton University, 1125 Colonel By Dr, Ottawa, ON K1S 5B6 Canada; 14 National Wildlife Research Centre, 1125 Colonel By Dr, Ottawa, ON K1S 5B6 Canada

**Keywords:** Arctic, Birds, Demography, Monitoring, Population trend, Tundra

## Abstract

Tundra-breeding birds face diverse conservation challenges, from accelerated rates of Arctic climate change to threats associated with highly migratory life histories. Here we summarise the status and trends of Arctic terrestrial birds (88 species, 228 subspecies or distinct flyway populations) across guilds/regions, derived from published sources, raw data or, in rare cases, expert opinion. We report long-term trends in vital rates (survival, reproduction) for the handful of species and regions for which these are available. Over half of all circumpolar Arctic wader taxa are declining (51% of 91 taxa with known trends) and almost half of all waterfowl are increasing (49% of 61 taxa); these opposing trends have fostered a shift in community composition in some locations. Declines were least prevalent in the African-Eurasian Flyway (29%), but similarly prevalent in the remaining three global flyways (44–54%). Widespread, and in some cases accelerating, declines underscore the urgent conservation needs faced by many Arctic terrestrial bird species.

**Electronic supplementary material:**

The online version of this article (10.1007/s13280-019-01308-5) contains supplementary material, which is available to authorised users.

## Introduction

Circumpolar Arctic tundra environments support a unique, and uniquely threatened, avifauna. Avian diversity declines with latitude, and is low compared to temperate and tropical regions (Gaston and Blackburn 2000). Only 2% of global bird species are known to breed regularly in the Arctic, and fewer still are Arctic specialists (Ganter and Gaston 2013). Although diversity is low overall, groups such as the geese (Anserini) and shorebirds (Scolopacidae, Charadriidae) achieve their highest diversity at Arctic latitudes, dominating the bird communities in many locations. More unique still is the highly migratory nature of these bird communities; nearly all Arctic-breeding bird species migrate to warmer regions during the non-breeding season, connecting the Arctic to all corners of the globe (Ganter and Gaston 2013).

Conservation threats are present throughout this globe-spanning range. In recent decades, the observed effects of climate change have been most pronounced at high latitudes (ACIA 2005; IPCC 2018), and in coming decades, the Arctic is predicted to experience warming twice that of the global average (Collins et al. 2013). Effects of rapid climate change are already measurably impacting tundra ecosystems, from a northward expansion of treeline (Harsch et al. 2009) or shrub habitats (Myers-Smith et al. 2011), to a drying of landscapes (Liljedahl et al. 2016), or a mismatch between the hatch of tundra birds’ chicks and the emergence of their arthropod prey (McKinnon et al. 2012; Senner et al. 2017). After the breeding season, Arctic-breeding birds fan out from boreal to tropical habitats, some travelling as far as the Antarctic. Throughout this broad range, they face many anthropogenic pressures, from modification and loss of habitats (e.g. loss of intertidal staging habitats in the Yellow Sea; Studds et al. 2017), to sport and subsistence harvest in flyways around the world (e.g. Reed et al. 2018).

The population status and trends of Arctic-breeding birds have been reviewed previously; however, widely differing efforts across species groups, regions and time periods (NABCI Canada 2012; Ganter and Gaston 2013; Deinet et al. 2015; this study) have reached different conclusions about the species and regions in need of greatest conservation attention. The Arctic Council’s Circumpolar Biodiversity Monitoring Program (CBMP; Petersen et al. 2004) was designed to harmonise data collection and synthesise results describing the status of biodiversity around the circumpolar Arctic. Birds are a conspicuous feature of this biodiversity, and because of their societal and ecological importance, they are identified as Focal Ecosystem Components for monitoring under the CBMP Terrestrial Monitoring Plan (Christensen et al. 2013).

Here, we review the status and trends of all terrestrial bird species around the circumpolar Arctic, reporting on trends in abundance, the most widely monitored metric. We examine variation in trends across four major global flyways, and across functional groups of birds, to identify regional or taxon-specific patterns in trends that suggest causation. We examine the quality of trend information that contributes to the population status of species around the circumpolar Arctic, and review the factors associated with declining trends. We comment on the urgency of the conservation issues facing terrestrial birds of the Arctic, and suggest that coordinated, international efforts to study and monitor populations are essential for understanding and ultimately reversing the alarming declines.

## Methods

### Study area

**Fig. 1:**
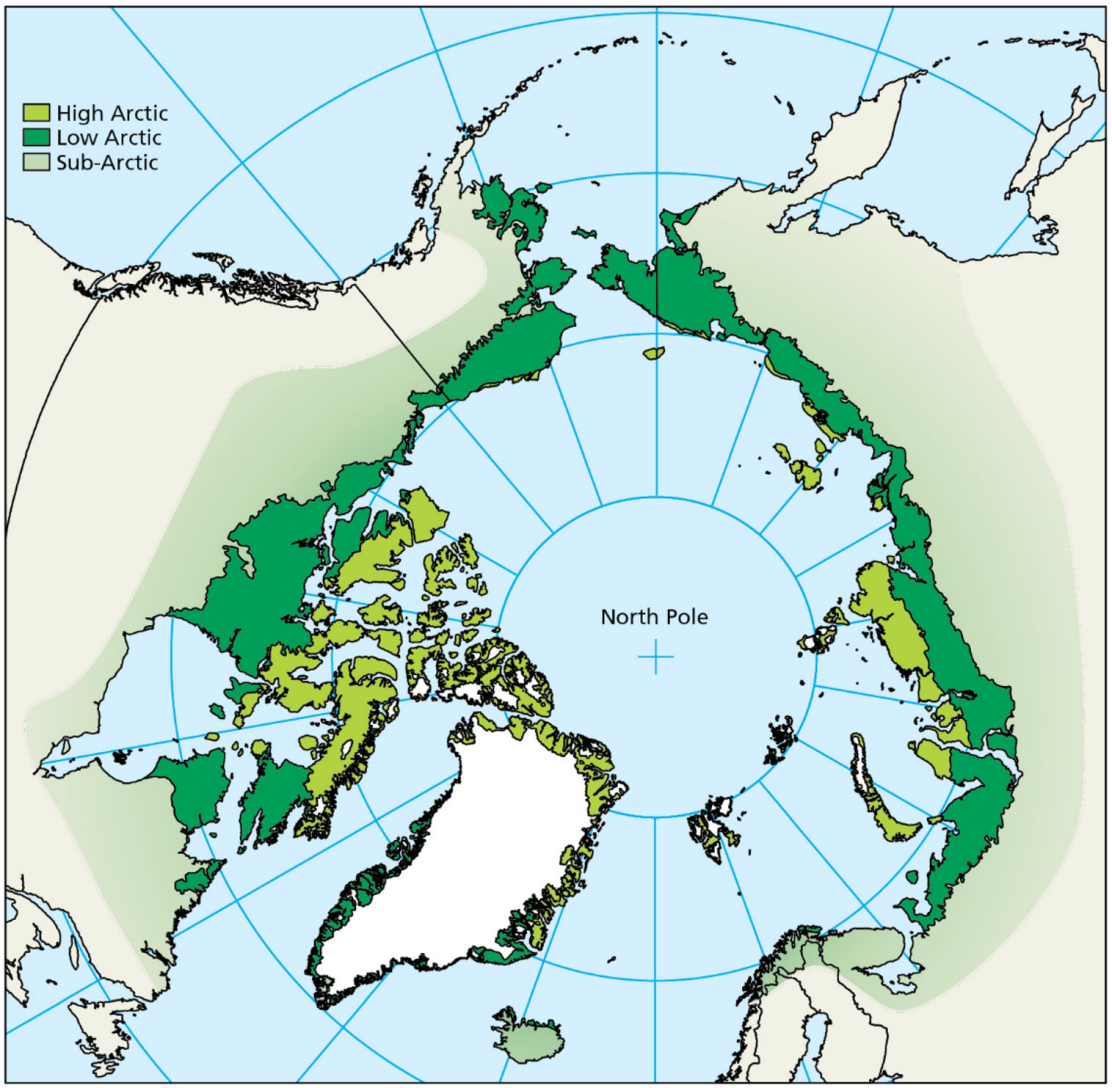
The study area for this assessment is the low and high Arctic, as outlined by the Circumpolar Arctic Vegetation Map (CAVM Team 2003) and the Arctic Biodiversity Assessment (Meltofte 2013). An approximation of the sub-Arctic is provided for reference (following Meltofte 2013), but species primarily inhabiting the sub-Arctic are excluded from analyses

Our assessment is focused on the high and low Arctic as defined in the Arctic Biodiversity Assessment (Meltofte 2013; Fig. 1). The distinction between high and low Arctic reflects the division between bioclimatic zones C and D in the Circumpolar Arctic Vegetation Map (CAVM Team 2003), on the basis of mean July Temp (5–7 °C vs. 7–9 °C) as well as primary production, plant species richness and vegetative communities. Population trends for birds in the sub-Arctic are not included in this assessment.

The Arctic comprises some 7.1 million km^2^ of terrestrial habitats, varying from the lightly to heavily vegetated shrub tundra of the low Arctic to the sparsely vegetated barrens that dominate in the northernmost parts of the high Arctic. This habitat variation is accompanied by a strong latitudinal gradient in primary production, with net annual production ten times lower in the islands of the high Arctic in comparison to the low Arctic (e.g. Bazilevich et al. 1997). Primary production also varies longitudinally, significantly greater in Arctic Alaska, coastal Russia west of Novaya Zemlya, and on the Taymyr Peninsula, than elsewhere (CAVM Team 2003).

**Fig. 2:**
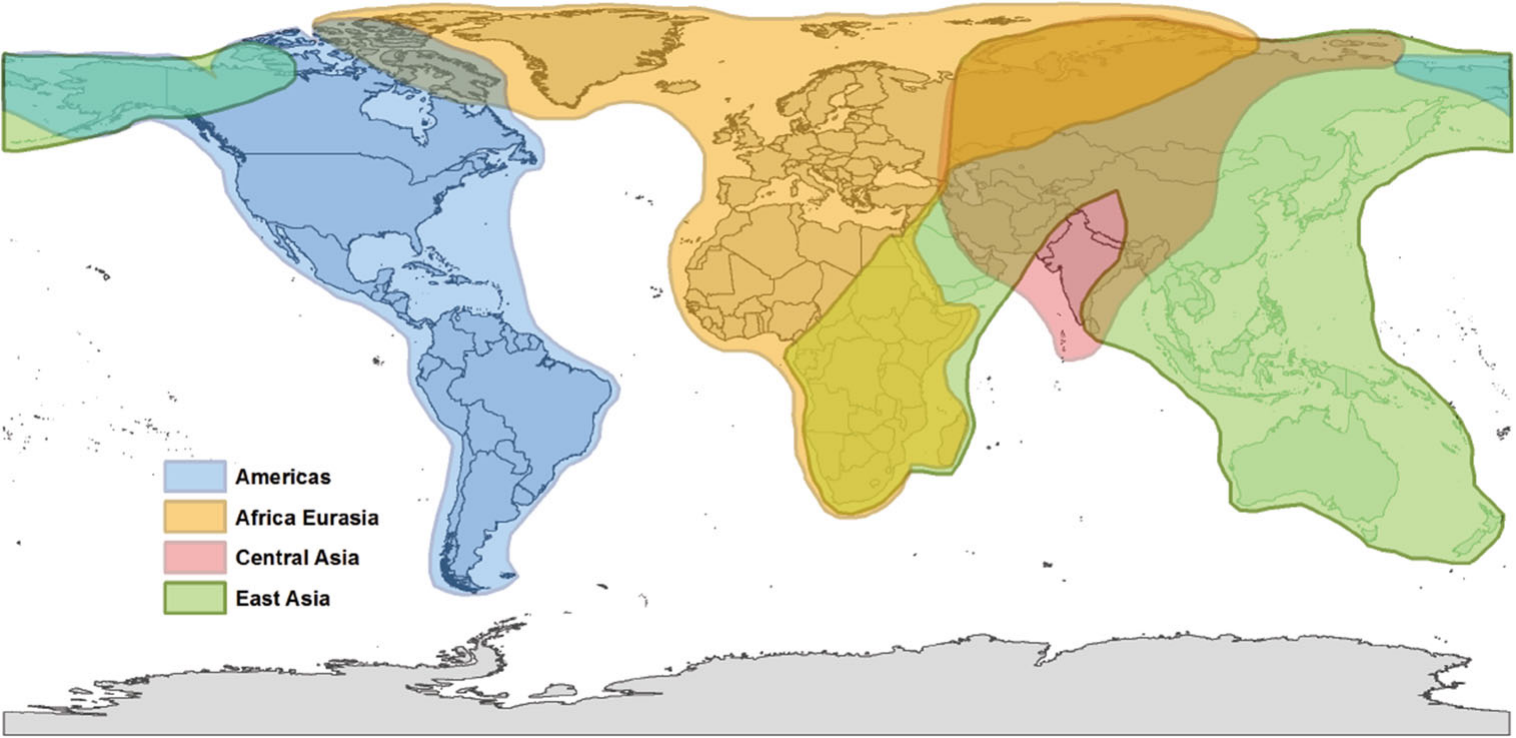
The four global flyways used in this assessment, from Deinet et al. (2015)

These biogeographic patterns in Arctic habitats significantly influence the diversity and abundance of birds (Ganter and Gaston 2013, see below). Distributions are also influenced by the routes birds take between breeding and non-breeding ranges, avoiding major ecological barriers and contributing to the emergence of distinct flyways. The term “flyway”, however, can have a variety of meanings that combine ecological and political boundaries, and differ with spatial scale (Boere and Stroud 2006). Here, for the sake of reporting patterns in status of birds at a global scale, we rely on four flyways (Fig. 2). Although finer resolution is possible in some regions, and ecologically appropriate for some species, this level of resolution allows reporting at a scale that matches global conservation agreements such as the African-Eurasian Waterbird Agreement, the East Asian-Australasian Flyway Partnership, or the North American Migratory Birds Convention.

### Species and metrics considered

We focus on terrestrial species that rely primarily on Arctic habitats for breeding. Approximately 200 species of birds are known to breed regularly in the Arctic, with 162 species considered to have 50% or more of their breeding range confined to the Arctic (i.e. Categories 1 and 2, and distinct Arctic subspecies of Category 3 species in Ganter and Gaston 2013). Of these, we considered 88 species to be terrestrial rather than marine (i.e. excluding seabirds and some seaducks); the status of Arctic marine birds is reported elsewhere (e.g. SAMBR: CAFF 2017). We further divided these 88 species into recognised subspecies or populations, following established species databases (e.g. Partners in Flight 2017; Fox and Leafloor 2018; Wetlands International 2018). This yielded 228 taxa for which status and trends were reviewed (Supplementary Data S1).

For some analyses, we grouped these species into guilds following the conventions of the Arctic Terrestrial Biodiversity Monitoring Plan (Christensen et al. 2013), namely insectivores (shorebirds/waders, passerines), carnivores (birds of prey, owls, jaegers/skuas), herbivores (geese, swans, ptarmigan), and omnivores (cranes, ducks, piscivores). These guilds are a simplification of the diet and behaviour of many species; for example “insectivores” eat many non-insect invertebrates, and many insectivorous species have omnivorous diets outside of the breeding season. However, the groupings are nonetheless useful to capture broad divisions in species’ contributions to ecosystem function. We report population trends for these guilds, as well as for taxonomic groupings (waterfowl, waders, other waterbirds, landbirds including passerines, raptors and ptarmigan) for comparison with previous assessments.

The Arctic Terrestrial Biodiversity Monitoring Plan (Christensen et al. 2013) identifies a wide range of desirable monitoring attributes related to birds, reflecting the full hierarchy of biodiversity from genes to communities. However, we presently do not monitor these with sufficient intensity for circumpolar reporting, in particular, lacking time series for finer-scale metrics such as genetic diversity. Christensen et al. (2013) identified the status and trends in abundance, demographic parameters and distribution for herbivores, carnivores and insectivores as having the highest priorities for monitoring. Here, we report on these priority measures of birds’ population status, highlighting important regional and taxa-specific gaps.

### Sources and quality of data

Data used to describe population sizes and trends were drawn from published and unpublished sources that varied greatly in temporal and spatial coverage. Sources per species are listed in Supplementary Data S1; important sources include Wetlands International’s database of Waterbird Population Estimates (Wetlands International 2018), the African-Eurasian Waterbird Agreement’s Conservation Status Review (AEWA-CSR7), BirdLife International’s database (BirdLife International 2018), North American analyses of trends from the Audubon Christmas Bird Count (Soykan et al. 2016), the International Shorebird Survey (PAS and ACS, unpubl.), a comprehensive review of population size and trends for waders in Australia (Hansen et al. 2016), and other regional- or taxa-specific sources. For geese, we used the recent global audit of goose populations (Fox and Leafloor 2018) and for birds of prey the recent review of Franke et al. (2020; this volume). These data were combined to produce a comprehensive database of species’ population sizes and trends, which was then reviewed and updated by taxonomic and regional experts to reflect the best available trend information for the species.

Population sizes and trends are presented largely following the conventions of Wetlands International’s Waterbird Population Estimates Database (Wetlands International 2018). Categorical trend estimates were assigned for long- (typically ≥ 30 years) and short-term periods (typically, 15 years), with data quality assessments of the estimates, from 1 to 4, reflecting unreliable or no supporting data (1) to international monitoring programs with defined statistical precision (4; see Supplementary Data S1 for additional details). Trend categories were “decline”, “stable”, “increase”, “fluctuating” or “unknown”. The stable category was assigned when trends were estimated to lie between − 0.64 to + 0.64% per year, equating to a 25% decrease or a 33% increase over a 45 year period, following the definition of stable for trend reporting exercises in North America (NABCI Canada 2012). These modest rates of decline or increase, while not trivial over long time scales, are often difficult to detect given the imprecision in many large-scale surveys. Where a single estimate of trend is reported, we used the trend (short- vs. long-term) based upon higher quality data. Where both were equally reliable, the long-term trend was favoured unless otherwise indicated.

The Arctic Shorebird Demographics Network provides valuable current demographic information, but no long-term trends. The International Breeding Conditions Survey on Arctic Birds (http://www.arcticbirds.net/; Tomkovich and Soloviev 2017) provides long-term data, but often only semi-quantitative. To our knowledge, there have been no long-term, multi-species reviews of distributional changes of Arctic birds for the circumpolar Arctic (but see Lappo et al. 2012). Reporting on these attributes takes the form of case studies, for regions where data exist.

### Analyses

Categorical estimates of trend are presented across flyways, guilds and taxonomic groupings. Recognising the qualitative nature of these status assessments, we kept analyses of these categorical data to a minimum. For North America, sufficient quantitative monitoring data existed to summarise trends using a multi-species index of abundance over time. A precision-weighted index was calculated across guilds/taxonomic groupings using a hierarchical Bayesian model described in Sauer and Link (2011), and as used in “State of the Birds” reports in Canada and the United States (e.g. NABCI Canada 2012). The model estimates the geometric mean population change with respect to a base year; we used 1980 as our base year because most surveys provided reliable estimates by this time. We calculated the population change since the base year as a ratio of population estimates or indices of population size for each species (Population_2016_/Population_1980_). Because imprecise estimates of population change tend to be extreme, the model accounts for the precision of each species’ estimated population change in calculating the group mean. For each year in the time series, the indices provide an estimate of the average population change since 1980 across all species in the guild or taxonomic group. The vertical axes of the index graphs (% change since 1980) are plotted on a log-scale to ensure that the visual representations of increases and decreases are comparable (e.g. so that a halving appears the same as a doubling).

## Results

### Global patterns in diversity and abundance

#### Insectivores

**Fig. 3:**
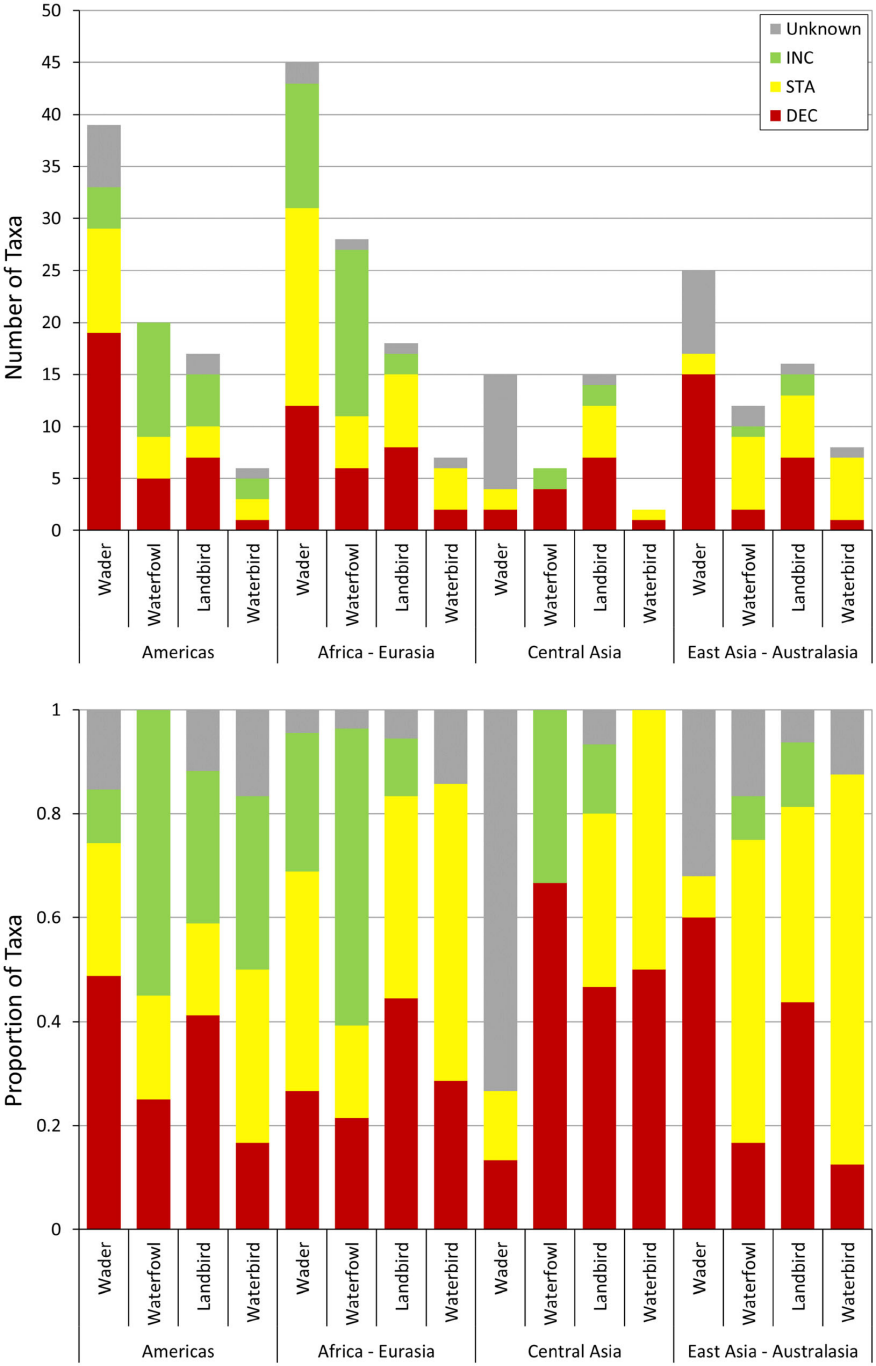
Trends in population abundance (increasing, stable, decreasing, unknown) for four taxonomic groupings of Arctic terrestrial bird species, in four broad global flyways. Trends are displayed as the total number of taxa (species or recognised populations; top), and as the proportion of the total taxa (bottom)

**Fig. 4:**
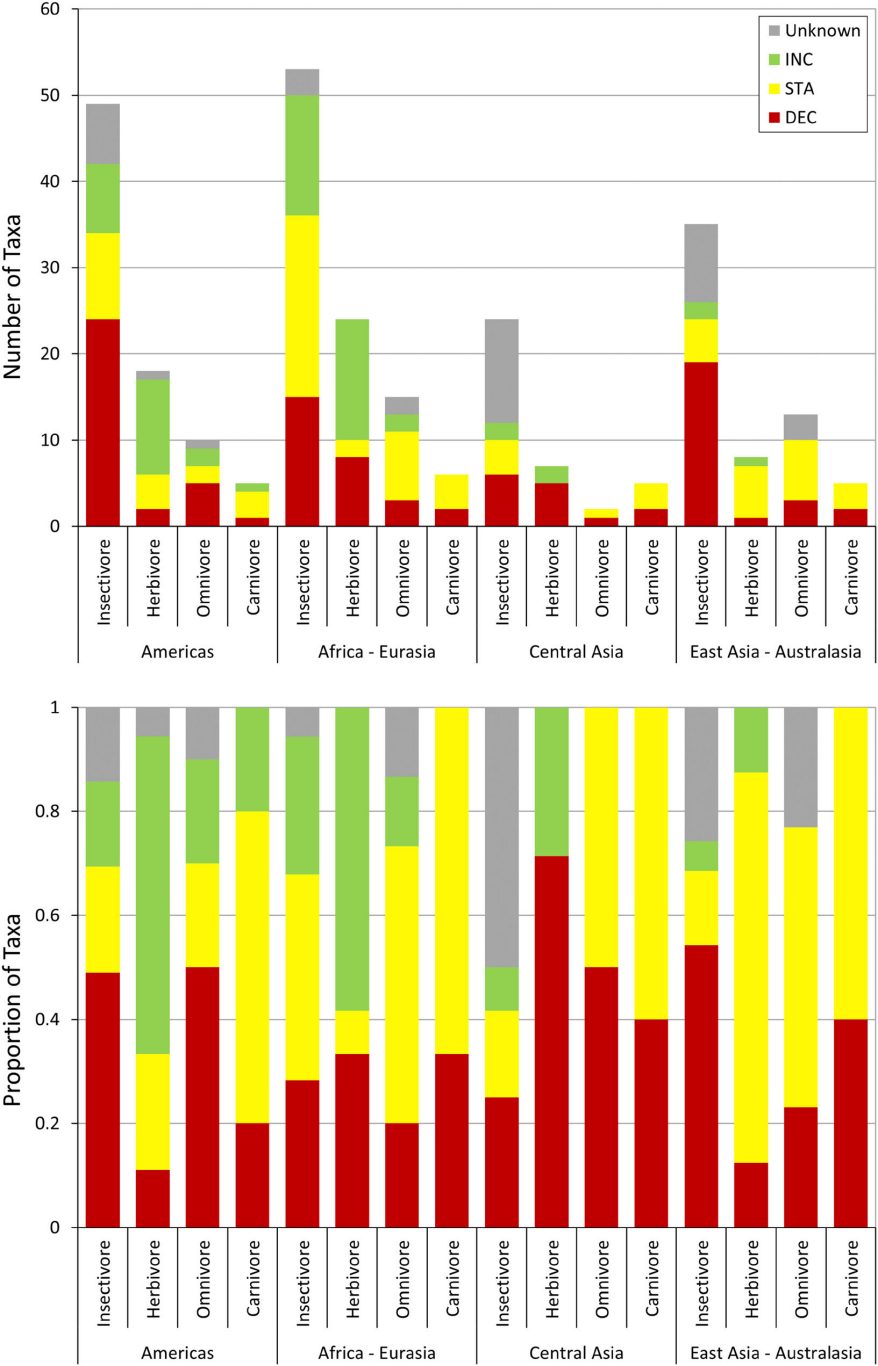
Trends in population abundance (increasing, stable, decreasing, unknown) for four guilds of Arctic terrestrial bird species, in four broad global flyways. Trends are displayed as the total number of taxa (species or recognised populations; top), and as the proportion of the total taxa (bottom)

At the species level, Arctic terrestrial birds are dominated by waders, comprising 41 of the 88 species (47%). This value is similar at the level of subspecies or populations, where waders represent 51% (117/228 taxa) of the diversity of terrestrial birds across the circumpolar Arctic. This dominance of wader subspecies richness is consistent across all but the Central Asian Flyway, where the wader and landbird group consists of the same number of taxa (15 each; Fig. 3). When waders and insectivorous landbirds are combined into an “insectivore” guild, the guild is clearly dominant across all flyways (Fig. 4). Within this guild, 49% of the taxa with known trends are believed to be declining.

Although the overall diversity of landbirds is low (30/228 taxa, or 13% across all flyways), some of these taxa are abundant and broadly distributed. Several small passerine species occur as a single taxonomic unit across the entire circumpolar Arctic, and the 5 largest population sizes all belong to passerines. The most abundant Arctic terrestrial bird, the Lapland longspur (*Calcarius lapponicus*), is estimated to have a global population size of perhaps 130 M individuals (Partners in Flight 2017), greater than the sum of the maximum population estimates for all non-passerines combined. For comparison, Arctic waders worldwide may number up to 50 million individuals (Zöckler 2012).

#### Herbivores

In Arctic tundra habitats, geese are by far the dominant herbivorous bird, with ptarmigan and tundra swans (*Cygnus columbianus*) being the remaining species. The status and trends of willow and rock ptarmigan (*Lagopus lagopus*, *L. mutus*) are reviewed in detail by Fuglei et al. (2019; *this volume*; trends are most often cyclical and vary widely across regions). The recently completed Northern Hemisphere Goose Audit suggested that there are 39.0–39.2 million wild geese belonging to 68 populations of 15 species (Fox and Leafloor 2018); 11 species and 42 of the management units are considered primarily Arctic tundra birds (Supplementary Data S1). These populations are distributed throughout all four flyways, with the greatest diversity in the African-Eurasian and Americas flyways.

The “white” geese (*Chen*) of North America are most numerous (17.1 million individuals of 3 species); all six populations have increased their abundance and distribution dramatically in the last 10 years, and several are listed as overabundant by management authorities in Canada and the United States (e.g. ECCC 2017). The Arctic taxa of “black” geese (*Branta*) number *c.*6.1 million individuals of 15 populations from 4 species, and all but one of these populations is stable or increasing over the long term. The Arctic “grey” geese (*Anser*) comprise 21 populations of 4 species, totalling approximately 6.4 million individuals, of which 7 populations have declined in abundance over the long term.

#### Carnivores

Only four birds of prey are considered true Arctic tundra species, and each is distributed broadly across the circumpolar Arctic. The peregrine falcon (*Falco peregrinus*) occurs as two distinct subspecies: the Arctic peregrine falcon (*F. p. tundrius*) in the Nearctic and the Siberian tundra peregrine (*F.p. calidus*) in the Russian Arctic. Both subspecies are highly migratory, with many individuals wintering south of the equator. The total number of adult breeding birds in the Nearctic is 32 000 individuals based on estimates from migration counts that include a proportion of the non-arctic subspecies *F. p. anatum* (Franke 2016). In the Palearctic, the *F. p. calidus* population is estimated at around 10 000 individuals, which is likely a significant underestimate (White et al. 2013).

The gyrfalcon (*Falco rusticolus*) has a circumpolar distribution with most adult birds resident in the North year-round (Potapov and Sale 2005). Approximately 8 000 gyrfalcon are found in the Nearctic and 12 000 in the Palearctic (Potapov and Sale 2005; Birdlife International 2018), with no significant long-term changes in population size reported (see Watson et al. 2011). The gyrfalcon is largely a prey specialist relying on ptarmigan, and their reproductive output is often linked to the cyclic abundance of ptarmigan.

The rough-legged hawk (*Buteo lagopus*) is the most abundant raptor in the Arctic, with a global population size in the range of 300 000 to 1 million mature individuals (Supplementary Data S1). Occupancy and productivity fluctuate with the cyclic abundance of their main prey, small mammals. In some areas (e.g. Scandinavia, Yamal) the population has declined dramatically since the 1970s, because of changes in the abundance or cyclicity of small mammals (Golovatin et al. 2010; Hellström 2014). Large-scale surveys of rough-legged hawks in northern temperate migration or wintering areas report high variability in abundance, but suggest a long-term decline (e.g. Kjellén 2018).

The snowy owl (*Bubo scandiacus*) is also a specialist on small mammals, with local breeding densities fluctuating widely in response to cyclic small mammal populations. Population size for this species has been the subject of debate, once estimated at 200 000 individuals but now believed to be as low as 8 000–28 000 individuals (Potapov and Sale 2013); long-term declines in this small population size have led to a recent IUCN classification of Vulnerable for this species (BirdLife International 2018). Like the gyrfalcon, some snowy owls remain in the Arctic during the non-breeding season, taking advantage of concentrations of seabirds in polynyas or other concentrations of prey (Therrien et al. 2011).

### Trends in abundance, all species

Trends in abundance differ greatly across taxonomic groupings and across flyways, as did the quality of trend information. Data were so poor that trends could not be estimated for 32 of 228 taxa, ranging from 5% (3/64) of waterfowl to 21% of waders (25/117). A single species (Eskimo curlew, *Numenius borealis*) is currently believed to be extinct, but not yet formally designated as such (COSEWIC 2009). Across all flyways, excluding taxa with unknown trends and the Eskimo curlew, declines were most prevalent in waders (51% of 91 taxa with estimated trends) and least prevalent in waterfowl (25% of 61 taxa). Conversely, increasing population trends were most common in waterfowl (49%) and least common in waders (15%) and other waterbirds (13% of 15 taxa).

These broad patterns were generally mirrored within flyways, with some exceptions. Within flyways, increases were generally most common in waterfowl and least common among waders and waterbirds (Fig. 3). Fewer waterfowl populations were increasing in the Central Asian and East Asian-Australasian flyways. The largest number of declining species was among the waders in all but the Central Asian flyway, where a large majority of waders had unknown trends. However, as a proportion of flyway taxa, stable or increasing trends dominated among waders in the African-Eurasian flyway, in contrast to other flyways (Fig. 3). These differences between regions were similarly pronounced for the insectivore guild (Fig. 4). Although diversity of waders was moderate in the East Asian-Australasian Flyway, fully 88% (15/17) of taxa with known trends were estimated to be declining; the largest proportion of any group.

Short- (most recent 15 years) and long-term (> 30 years) trends were available for 157 taxa. Trends were unchanged over the two time periods for a majority of taxa (80%), improved for 11% of taxa and worsened for 9% of taxa. Differences among species groups were minor, with the percentage of worsening trends ranging from 0% for landbirds and waterbirds to 14% for waders.

**Fig. 5:**
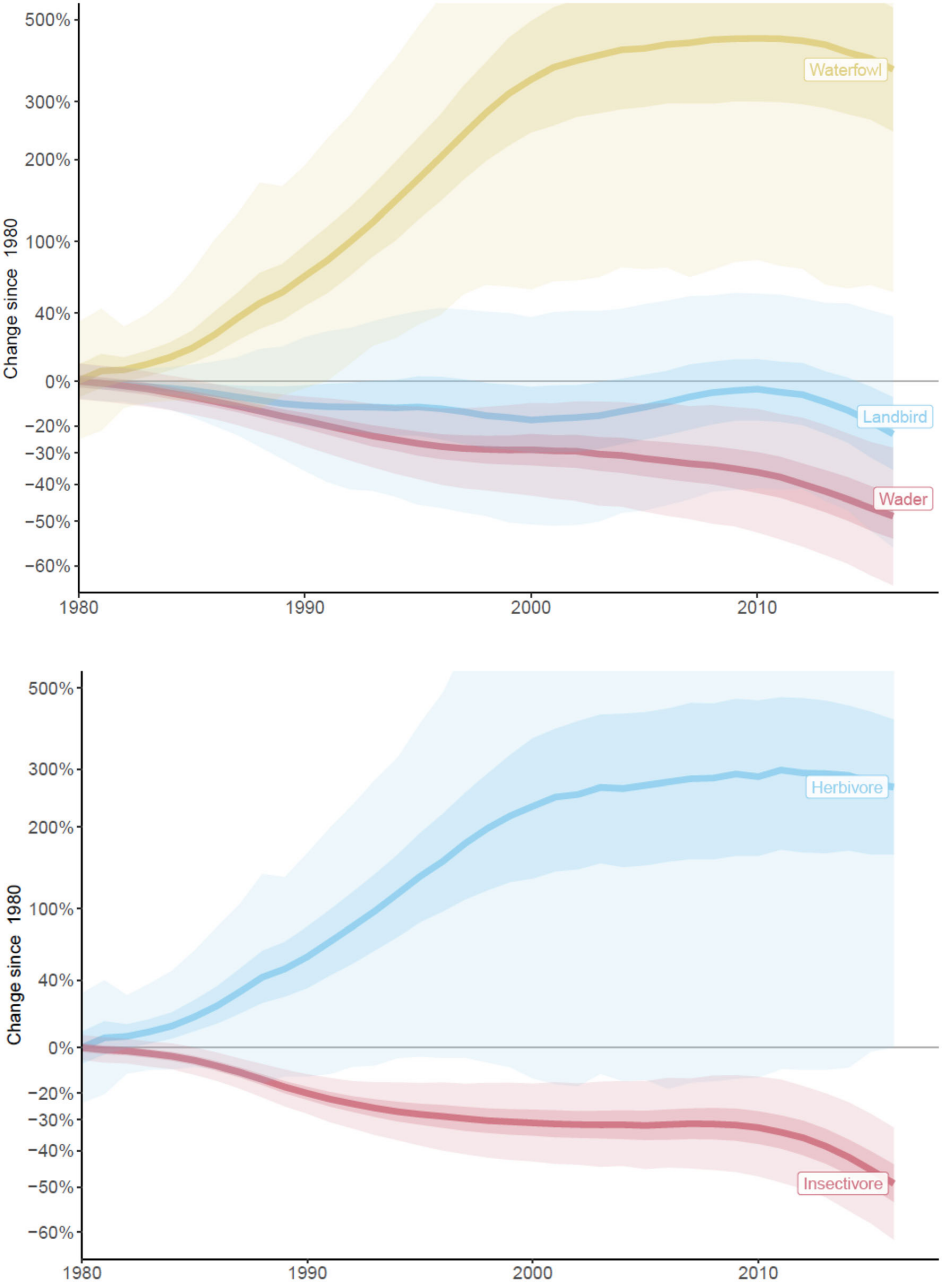
Composite indices of trends in abundance for North American Arctic birds of various taxonomic groupings, 1980–2016. Light and dark shading denote 50% and 95% credible intervals, respectively. Groupings reflect standard taxonomic divisions (top) or foraging guilds (bottom). The following groupings were omitted owing to small sample sizes (< 5 taxa): waterbirds (such as loons and jaegers; top), omnivores, carnivores (bottom)

Within North America, continuous time series of monitoring data for most waterfowl and waders permitted the calculation of quantitative indices of trend. In contrast, waterbird, omnivore and carnivore groups had too few species (< 5) to calculate indices reliably. Arctic-breeding waterfowl tripled in abundance relative to the 1980s, largely as a function of increases in white geese, while Arctic-breeding waders halved in abundance and landbirds declined by ~ 20%. All groups, including waterfowl or herbivores showed a tendency towards accelerating declines in the last decade, with an inflection around 2010 (Fig. 5). While concerning, the cause of these coincident trends remains unclear and warrants further investigation.

### Data quality

**Fig. 6:**
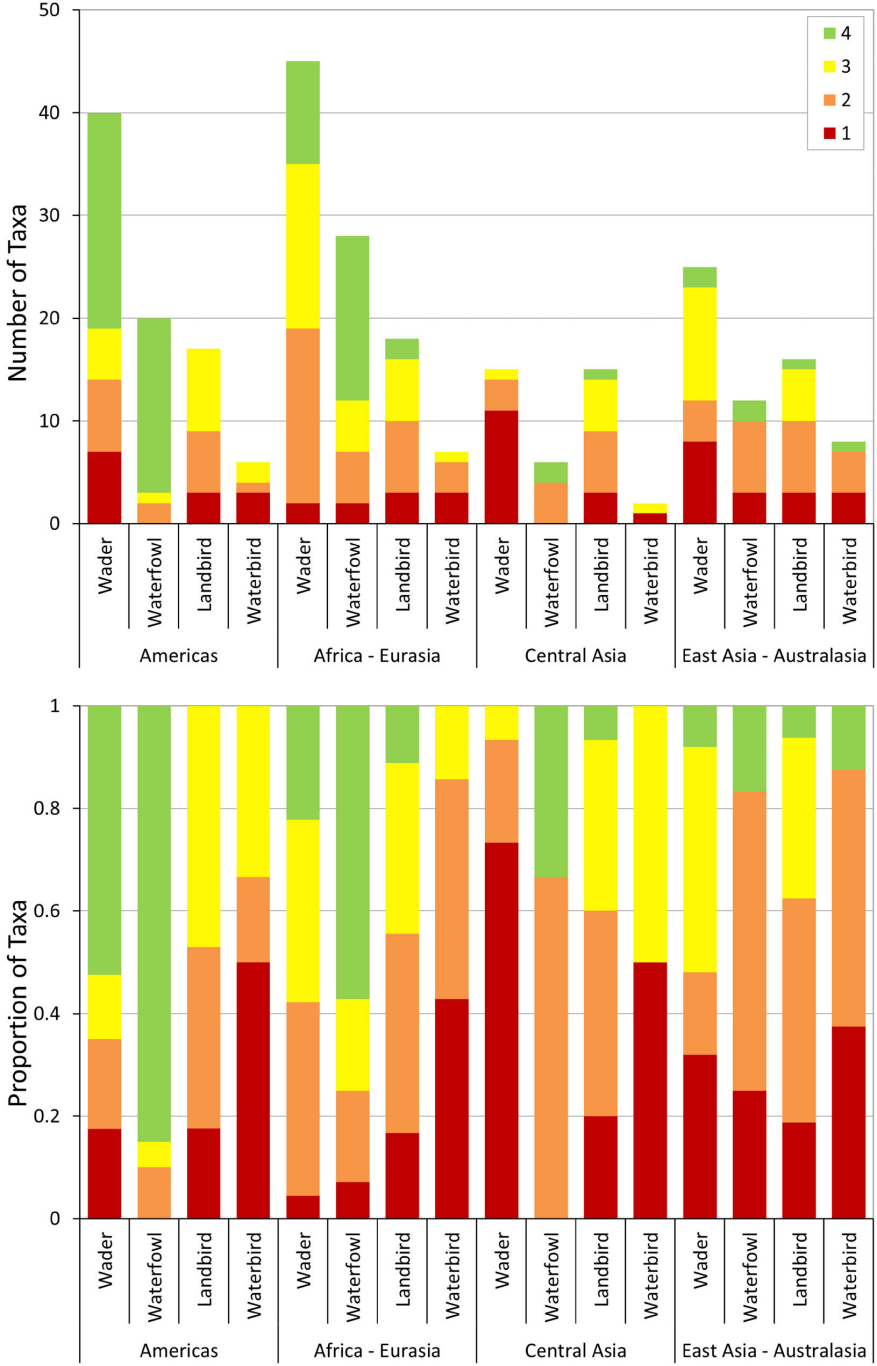
Quality of the monitoring information used to describe trends in abundance for the number (top) and proportion (bottom) of populations within taxonomic bird groups in four global flyways. Trend quality categories are as follows: (1) data are lacking such that trends are unknown, (2) regional and site-specific monitoring allow for assumptions of trend, (3) international monitoring allows estimation of trend direction, and (4) rigorously designed international monitoring programs yield estimates of precision

The quality of the monitoring data documenting trends in population abundance varied widely among regions and taxonomic groups (Fig. 6). Trend data were lacking altogether, for any time period, for 32 of 228 taxa (14%, trend quality = 1; Supplementary Data S1). In all flyways, waterfowl and waders had the highest quality monitoring data, with a mean (± SD) trend quality of 3.2 ± 1.1 and 2.6 ± 1.1, respectively, in comparison to 2.4 ± 0.8 for landbirds, and 2.1 ± 0.9 for waterbirds (scores range from 1 to 4). The quality of monitoring in the Americas flyway (3.0 ± 1.1) and the African-Eurasian flyway (2.8 ± 1.0) was better than in the East Asian-Australasian (2.2 ± 1.0) or Central Asian (1.9 ± 1.0) flyways. The quality of monitoring information was similar for declining species (2.95 ± 0.8) versus stable or increasing species (2.99 ± 0.97), offering no evidence that experts were biased in their willingness to conclude declines versus increases when confronted with poor data.

Importantly, the proportion of populations lacking trend estimates for the most recent 15 years (25%) was similar to the proportion over the long term (19%), with a similar mean trend quality in both periods (2.5 ± 1.1 and 2.6 ± 1.1, respectively). These facts suggest that monitoring programs are neither more comprehensive nor more rigorous in recent years than previously (Supplementary Data S1).

## Discussion

### Biogeographical patterns in density and diversity

Avian diversity generally follows gradients in productivity, with higher diversity in the low vs. high Arctic, peaking in Beringia and lowest in Greenland and Svalbard (Zöckler 1998; Ganter and Gaston 2013). The resolution of our global flyways, and their differing sizes, obscures this relationship to some extent, with highest diversity in our dataset in the two largest flyways: the Americas and Africa-Eurasia. Within the Americas flyway, tundra bird diversity is greater in Alaska than in Arctic Canada, and wader densities are > 5 times greater in parts of Arctic Alaska than Arctic Canada (Bart and Smith 2012).

Even within these regions, density and diversity respond to productivity gradients. Heavily vegetated wetlands dominated by sedges, grasses and mosses constitute a small fraction of the total land area in many regions of the circumpolar Arctic, but hold a disproportionate richness and abundance of birds. Although wetlands constituted only c. 20% of Arctic North American vegetated habitats, they held nearly 50% of waders, owing to 2.5–5 times greater diversity and nesting densities there (Bart and Smith 2012). Hence, circumpolar tundra bird abundance and diversity is highly concentrated in the most productive habitats.

### Correlates of trend

#### Insectivores

Waders dominate the insectivore guild in species numbers, and emerged as a conservation priority throughout the results, showing the largest proportions of declining species of any taxonomic group, with the greatest proportion of declining taxa in the East Asian-Australasian Flyway. The dramatic declines of waders in this flyway are thought to relate to a > 65% loss of intertidal habitat in the Yellow Sea (e.g. Studds et al. 2017). Proportions of species’ populations staging in the Yellow Sea was the strongest predictor of population trend, suggesting that failure to accrue sufficient resources during staging impacted a birds’ survival post departure (Studds et al. 2017). Similarly, in the West Atlantic flyway, individuals of the endangered *rufa* red knot (*Calidris canutus rufa*) have been shown to have reduced migratory performance and survival when they depart the primary staging site, Delaware Bay, USA, in poor body condition, as a consequence of reduced availability of their preferred forage, horseshoe crab eggs (*Limulus polyphemus*; Baker et al. 2004; Duijns et al. 2017). In contrast, in the East Atlantic portion of the African-Eurasian flyway, where there are more stable and positive wader trends than elsewhere, some have suggested a link to increased forage availability from eutrophication of coastal staging habitats (Meltofte et al. 2019).

These examples demonstrate the crucial importance of conditions at migratory staging sites for Arctic waders, most of which are long-distance migrants. The impacts of poor energetic status during migration may be particularly acute for individuals that migrate over the open ocean or other ecological barriers, with limited ability to stop safely *en route*. The large proportion of declining species in the East Asian-Australasian and Americas flyways could reflect a high proportion of oceanic migrants.

Poor energetic status of birds due to the loss or degradation of migratory staging sites could also affect future reproductive success (e.g. Senner et al. 2014; but see Duijns et al. 2017; ). Reproductive success of Arctic tundra waders is highly variable in space and time for many reasons, for example increasing with latitude (McKinnon et al. 2010; but see Bulla et al. 2019), or varying with snow conditions, weather, or variation in predator abundance and their preferred small mammal prey (reviewed in Meltofte et al. 2007). Changing environmental conditions in the Arctic could help to explain why declines exist in some species but not others in flyways where staging sites have not declined radically in quality in recent years, such as in the East Atlantic portions of the African-Eurasian Flyway. However, quantitative evidence to link Arctic environmental change to population declines remains limited (see “Climate-related Mismatch as a Stressor”).

Although waders dominate tundra habitats in species richness, passerine landbirds clearly dominate numerically. Most of these abundant and broadly distributed species, several with circumpolar distributions, appear to show long-term increases in abundance. However, continuous time series from North America suggest declines in the last decade, consistent with observations of declines in some portions of Russia (Supplementary Data S1). Despite their ubiquity, the quality of trend information for these species is poor, partly due to difficulties in combining regional trend estimates across their broad and contiguous geographic ranges.

#### Herbivores

Most populations of geese showed increasing or stable trends over the last 30 years, but our knowledge of trends is highly variable (Zöckler 2008). North American population estimates are of the best quality and most populations are increasing or stable, as are those in Europe, probably due to increasing exploitation of agricultural landscapes for food outside of the breeding season (Fox and Abraham 2017). In Arctic North America, populations have increased to such an extent that they are adversely affecting their staging and breeding habitats through intensive grazing of graminoids, and “grubbing” of the below ground rhizomes, in some cases leading to lasting vegetation loss and habitat degradation (Abraham et al. 2012; Mariash et al. 2018), with evidence emerging of adverse density-dependent consequences for the geese themselves (e.g. Ross et al. 2018). Similarly, recent studies suggest that hyperabundant geese could have adverse effects on the density, habitat selection or reproductive success of sympatric tundra bird species at local and regional scales (e.g. Flemming et al. 2016, 2019).

In contrast, many goose populations are declining in Central and Eastern Asia, especially where species are confined to natural habitats of declining quality (e.g. Yu et al. 2017). However, good population estimates and count data series are generally lacking, except in Korea and Japan where many goose populations are associated with farmlands and are known to be increasing through coordinated surveys (Jia et al. 2016). The quality of survey data is rapidly improving in China, where count networks and coordination with flyway partners are being established (e.g. Zhao et al. 2012).

Although waterfowl had the highest quality monitoring data for any taxonomic group in our analyses, important gaps remain. A recent international review identified the most urgent of these needs, including improved delineation of flyway populations, more regular reporting of annual changes in abundance for all northern hemisphere goose populations, the initiation of demographic monitoring and research programs to identify causes of decline, and evaluation of the potential adverse effects of overabundant goose populations on habitats and sympatric species (Fox and Leafloor 2018).

#### Carnivores

Many peregrine falcon populations (especially in Alaska, parts of Canada and northern Fennoscandia) have now largely recovered from dramatic reductions due to pesticide contamination during the 1970s (Franke et al. 2020; this volume). Overall, Arctic peregrine populations now appear stable, with reports of a northward expansion among peregrines in Northwest Greenland (Burnham et al. 2012), as well as an advancing timing of breeding in several Arctic regions (Franke et al. 2020), likely as a consequence of climate change. Gyrfalcon populations are also stable, although poaching is an increasing concern in some areas (Potapov 2011), and shrubification and a shrinking High Arctic climate zone may pose challenges in the future, mediated through impacts on their primary prey, ptarmigan (Booms et al. 2011). The iconic snowy owl is believed to be declining range-wide, and was recently uplisted by IUCN to a status of vulnerable (Birdlife International 2018). Population trends for this lemming specialist are believed to be influenced in part by declines in the abundance of lemmings (*Dicrostonyx* spp., *Lemmus* spp.). Declines in lemmings could adversely affect other lemming predators as well, such as pomarine and long-tailed skua (*Stercorarius pomarinus*, *S. longicaudus*), both of which have declined dramatically in western Siberia (Golovatin et al. 2010). However, the evidence for declines in lemming abundance at a circumpolar scale is mixed (Ehrich et al. 2020, *this volume*).

### Climate-related mismatch as a stressor

Nearly all Arctic-breeding birds are migratory (Ganter and Gaston 2013), and the timing of their life histories has evolved in response to environmental cues across hemispheric scales. The accelerated rate of climate change at high latitudes (e.g. IPCC 2018) can potentially decouple such cues, leading to mismatches. Indeed, climate-related mismatches are widely proposed as among the leading stressors of wildlife populations arising from anthropogenic climate change (e.g. Both et al. 2006; Gilg et al. 2012). For herbivores, mismatched timing of breeding can impair chick growth because of a reduced nutrient content of forage plants later in the growing season (e.g. Clausen and Clausen 2013; Ross et al. 2018). Insectivorous species, too, could suffer due to phenological mismatch.

Arctic-nesting waders, for example, travel thousands of kilometres each spring to take advantage of a burst of arthropod prey during the Arctic summer. A phenological mismatch between the timing of reproduction and the period during which these arthropods are abundant is one of the key hypothesised effects of climate change on Arctic insectivores (Tulp and Schekkerman 2008; Saalfeld and Lanctot 2017), with some evidence of reduced growth rates of chicks due to mismatch (McKinnon et al. 2012), and reduced body size of juvenile red knots during years of early snowmelt in high Arctic Siberia (van Gils et al. 2016). However, there is considerable disagreement among studies; Hudsonian godwits (*Limosa haemastica*) in Alaska remain appropriately timed with respect to arthropods (Senner et al. 2017), sanderling (*Calidris alba*) chicks in Greenland have not been affected by the apparent mismatch documented there (Reneerkens et al. 2016), and there is evidence that temperature increases can alleviate some of the negative effects of phenological mismatch for waders via reduced thermoregulation costs (McKinnon et al. 2013), although such thermoregulatory benefits might be minor (Machín et al. 2018). Overall, despite the hypothesised importance of mismatch, there is considerable variation both in the extent of mismatch across species, and in the strength of evidence for its effects, perhaps due to variable influence of local environmental drivers across study sites, or life-history traits across species.

### Climate effects on distribution

A northward shift in climatic conditions and habitats is expected to result in a corresponding shift in the range of Arctic species, which for high-latitude avian species can result in an expansion of suitable climatic conditions (e.g. Zöckler and Lysenko 2000; Jensen et al. 2008), or an “arctic squeeze”, as suitable conditions are pushed off the edge of the map (Meltofte 2013; Wauchope et al. 2017). Numerous examples of changes in range have been reported for individual species of Arctic birds (*reviewed in* Ganter and Gaston 2013; Lappo et al. 2012), but syntheses of large-scale data to document range shifts across suites of species remain exceedingly rare. Large-scale “bird atlas” surveys in Finland allowed researchers to document a northward shift in range of 0.7 km/year for northern bird species between the 1970s and 2000s, and similar national atlases allow for similar analyses in Sweden and Norway (e.g. Elmhagen et al. 2015). Although a northward expansion of trees and shrubs is well documented (e.g. Harsch et al. 2009; Myers-Smith et al. 2011), large-scale and consistent monitoring schemes to track distributional changes in birds are lacking from the North American and Russian Arctic, making similarly rigorous analyses impossible.

### The importance of long-term and large-scale studies

This synthesis highlights that consistent monitoring, at large spatial scales and over long time periods, can play a crucial role in our understanding of the status and trends of Arctic bird populations. Some of these consistent monitoring efforts have arisen from collaborative research projects designed to address specific questions. For example, the Arctic Shorebird Demographics Network (Lanctot et al. 2015) sought to estimate shorebird survival and reproductive success across the North American Arctic (and parts of Russia) to determine the factors limiting populations (e.g. Weiser et al. 2018). The recently established “Interactions Working Group” is exploring predator–prey interactions by implementing standardised protocols across a network of sites spanning the circumpolar Arctic. Other programs bring together pre-existing datasets for large-scale, post hoc syntheses. For example, the International Breeding Conditions Survey on Arctic Birds (http://www.arcticbirds.net/; Tomkovich and Soloviev 2017) gathers circumpolar site-specific data describing Arctic environmental conditions, reproductive success, and bird abundance and diversity. During the non-breeding season, existing surveys of juvenile ratios and band resighting could provide improved demographic information for many species if analysed and reported in a coordinated fashion (Robinson et al. 2005). At even larger spatial scales, novel analytic approaches have been used to link demographic and phenological variables across entire flyways to understand when, where and how populations are limited (e.g. Rakhimberdiev et al. 2018). Such collaborative projects all draw primarily on existing field programs, adding value to these programs by facilitating large-scale analyses through the shared application of consistent monitoring and research protocols.

This existing research and monitoring infrastructure in the Arctic plays an even more crucial role in our understanding of the status and trends of Arctic birds and ecosystems. Most parts of the Arctic are remote, challenging and expensive regions in which to work, and the long-term maintenance of this infrastructure is both essential and challenging. Although many bird populations can be monitored outside of the Arctic, understanding status and trends in demographic parameters requires work on the Arctic-breeding grounds. Long-term field stations have also played an essential role in developing our understanding of Arctic ecosystem dynamics and responses to environmental change. For example, work at Zackenberg, Greenland, Bylot Island, Canada, and the Taimyr Peninsula, Russia, have transformed our understanding of interactions between tundra birds and other ecosystem components (e.g. Wirta et al. 2015; Reneerkens et al. 2016; Schmidt et al. 2017). National or international programs to maintain this field infrastructure through promotion of its importance, such as the European Union’s INTERACT program (https://eu-interact.org/), play an important role in maintaining the integrity of monitoring programs for tundra birds into the future.

### Knowledge gaps identified

Despite our best efforts to monitor birds throughout the Arctic, important data gaps remain. We demonstrate here that nearly half of all populations of Arctic tundra birds have monitoring information that is considered “poor” or worse. In the Central Asian and East Asian-Australasian flyways, less than one-third of populations exceed this modest standard for data quality. Some of the best datasets in the Americas and African-Eurasian flyways are available in part to support international obligations, such as the shared management of harvested waterfowl in North America under the Migratory Bird Treaty or the reporting obligations under the Agreement on the Conservation of African-Eurasian Migratory Waterbirds (AEWA). International partnerships are developing in other flyways, such as the East Asian-Australasian Flyway Partnership (EAAFP), and could enhance the availability of monitoring data in the future. Also, systematic efforts to collect and synthesise data from local- and regional sources to produce broad-scale indices, such as the Arctic Species Trend Index (Deinet et al. 2015), can ensure that we make the best possible use of the data that are collected. Importantly, however, we show that the quality of monitoring information has not yet improved when comparing trends over the last 15 years with trends over longer-time periods.

Information describing status and trends in demographic parameters is far more fragmentary, and is lacking for the majority of species in even the best monitored flyways. Improved understanding of demography is essential if we are to achieve an understanding of the causes of decline, which are likely multi-faceted, species-specific, and occurring both within and outside of the Arctic. For some species, such as the widespread Arctic passerines, even population structure is poorly defined, making regional gaps in monitoring difficult to identify. Addressing these gaps and improving understanding of the status and trends for Arctic tundra birds requires cooperation that is sustained over decadal time scales, spans the circumpolar Arctic and the global ranges of its migratory species, and brings together datasets in novel, integrative ways. However daunting, this collaborative monitoring is essential if we are to understand and ultimately reverse the declines of Arctic tundra birds.

## Electronic supplementary material

Below is the link to the electronic supplementary material.

Supplementary material 1 (XLSX 73 kb)
